# Supplementary material for: Brow and Masticatory Muscle Activity Senses Subjective Hedonic Experiences during Food Consumption
Source: Nutrients. 2021 Nov 24;13(12):4216. doi: 10.3390/nu13124216 (PMC8708739; doi:10.3390/nu13124216)
Supplement: Supplementary file 1 [file nutrients-13-04216-s001.zip › nutrients-1443921-supplementary.pdf]

nutrients

MDPI

Table S1. Mean (with *SD*) subjective ratings.

Stimulus	Measure			
	Liking	Wanting	Valence	Arousal
Vanillin	6.5	6.1	6.6	5.8
	(1.3)	(1.6)	(1.2)	(0.9)
Maltol	6.4	6.3	6.5	5.6
	(1.2)	(1.2)	(1.0)	(1.3)
Ethyl butyrate	6.5	6.2	6.5	5.9
	(1.4)	(1.9)	(1.3)	(1.1)
Phenethyl alcohol	4.4	3.9	4.3	5.0
	(2.0)	(1.9)	(2.0)	(1.8)
Acetoin	4.8	4.6	4.8	5.1
	(1.8)	(1.7)	(1.7)	(1.5)
2,5-dimethylpyrazine	3.6	3.2	3.4	5.1
	(1.5)	(1.5)	(1.5)	(1.9)
Isovaleric acid	3.9	3.4	3.7	4.9
	(1.6)	(1.7)	(1.5)	(1.8)
(E)-2-Nonenal	2.8	2.5	2.6	5.5
	(1.6)	(1.5)	(1.6)	(2.0)
Indole	3.7	3.2	3.8	5.0
	(1.7)	(1.7)	(1.7)	(2.0)

Table S2. Mean (with *SD*) physiological activity.

Stimulus	Measure						
	Corrugator	Zygomatic	Masseter	Suprahyoid	SCR	HR	Nose
Vanillin	−0.4	−0.1	0.0	0.1	−0.1	0.1	0.0
	(0.5)	(0.4)	(0.6)	(0.6)	(0.6)	(0.7)	(0.6)
Maltol	−0.1	0.1	0.1	0.1	0.0	0.1	0.3
	(0.7)	(0.5)	(0.5)	(0.6)	(0.7)	(0.6)	(0.8)
Ethyl butyrate	0.0	0.2	0.4	0.3	0.1	−0.2	−0.2
	(0.6)	(0.8)	(0.8)	(0.5)	(0.6)	(0.8)	(0.6)
Phenethyl alcohol	−0.1	0.0	−0.1	0.1	0.0	0.1	−0.2
	(0.6)	(0.6)	(0.6)	(0.5)	(0.5)	(0.7)	(0.8)
Acetoin	0.0	0.0	−0.1	0.0	0.0	0.1	0.2
	(0.6)	(0.5)	(0.6)	(0.6)	(0.7)	(0.7)	(0.7)
2,5-dimethylpyrazine	0.2	0.0	0.1	−0.1	−0.1	0.0	−0.2
	(0.6)	(0.7)	(0.7)	(0.6)	(0.6)	(0.6)	(0.7)
Isovaleric acid	−0.1	−0.1	−0.2	−0.1	−0.1	−0.2	0.1
	(0.7)	(0.7)	(0.6)	(0.7)	(0.7)	(0.8)	(0.5)
(E)-2-Nonenal	0.2	−0.2	−0.2	−0.1	0.3	0.1	0.1
	(0.7)	(0.8)	(0.8)	(0.7)	(0.8)	(0.7)	(0.8)
Indole	0.2	0.0	−0.1	−0.1	0.0	−0.1	0.1
	(0.8)	(0.7)	(0.7)	(0.9)	(0.6)	(0.6)	(0.8)

All physiological data were standardized within each individual. Corrugator = Corrugator supercilii; Zygomatic = zygomatic major; SCR = skin conductance response; HR = heart rate; Nose = Nose-tip temperature.
